# Special Issue “Application of NMR Spectroscopy in Biomolecules”

**DOI:** 10.3390/ijms27093776

**Published:** 2026-04-23

**Authors:** Luigi Russo

**Affiliations:** Department of Environmental, Biological and Pharmaceutical Sciences and Technologies, University of Campania “L. Vanvitelli”, 81100 Caserta, Italy; luigi.russo2@unicampania.it

Nuclear Magnetic Resonance (NMR) spectroscopy is a powerful technique to study the structural and dynamical peculiarities of small molecules and biomolecules such as peptides, proteins, and nucleic acids [1,2,3,4,5]. The continuous development of NMR methods, together with innovative isotope labeling strategies (^15^N, ^13^C, ^2^H, and ^19^F), has significantly improved the size limitation caused by slower tumbling rates and shorter NMR signal relaxation times of larger molecules [6]. NMR is not only a technique capable of solving protein structures at atomic resolution, but also offers different methodologies for describing motions in biomolecules across a wide range of timescales, from picoseconds to seconds, which are fundamental for folding, function, and binding [7,8,9,10,11,12]. In the last decade, solution NMR emerged as a particularly important experimental method to probe protein folding at atomic resolution, providing insight into kinetics, thermodynamics, and misfolded intermediate states [8,13,14,15,16]. Furthermore, NMR has a vast range of experimental techniques allowing the study of protein–protein, protein–ligand, or protein–DNA/RNA interactions in solution, on the cell membrane (on-cell), or inside living cells (in-cell) [17,18,19,20,21,22]. One of the advantages of NMR over current structural methods is that it can detect transient and weak intramolecular and intermolecular interactions in folded proteins [23] or even in disordered proteins/peptides (IDPs) [24,25,26,27] that are often crucial for modulating their structure, dynamics, and function. Finally, during the past few decades, NMR has been extended to the analysis of complex mixtures, such as biological samples (NMR-based metabolomics), including the quantification of metabolites (qNMR) [28,29,30].

The intention of this Special Issue was to highlight the new advances in technical developments and applications of solution and solid-state NMR methodologies in biology, pathology, and pharmacology, covering structural biology. This Special Issue contains a mixture of original articles, including experimental and methodological studies, shedding light on the current NMR capabilities in elucidating, at atomic resolution, the structural and dynamical determinants driving the functional behavior of proteins, peptides, and small molecules.

The Special Issue presents two methodological articles focused on the design of new pulse sequences for applications in the field of structural biology and on the development of an alternative approach for qNMR. Specifically, Piccioli M. and co-workers (contribution 1) present the development and application of innovative NMR methodologies based on the combination of high magnetic fields and optimal control pulses for studying the structural peculiarities of paramagnetic macromolecules, whereas the contribution by Shan and co-workers (contribution 4) reports a novel approach for qNMR based on ^1^H and ^19^F NMR, demonstrating that the proposed strategy is a fast, accurate, and simple method for performing quantitative analysis of compounds in solution.

Regarding NMR applications in the field of small molecules and metabolomics, the Special Issue contains three contributions: Gorbin S. I. et al. (contribution 9) use ^1^H, ^13^C, and ^31^P NMR spectra to characterize the structure of a novel synthesized compound, 1-[aryl-(diphenylphosphono)methyl]-3,4,6-trimethylglycolurils, obtained via the interaction of benzaldehyde and its mononitro- and monohydroxyderivatives with 1,3,4-trimethylglycoluril and triphenylphosphite; Drzewiecka D. et al. (contribution 2) report a serological and chemical study of the O-specific polysaccharide (OPS or O antigen) from *P. mirabilis* Bprz 86 LPS, isolated from a wound. In particular, a series of 1D and 2D NMR experiments were used to perform an accurate structural characterization of the six-constituent OPS repeating unit; Coronado M. A. and co-workers (contribution 7) report a metabolomic study based on the application of HR-MAS NMR (High Resolution Magic Angle Spinning Nuclear Magnetic Resonance) spectroscopy. The latter has been applied to explore the inhibitory potential of wedelolactone against the nonstructural protein 2 of CHIKV, as well as the metabolic disturbances in model mammalian cultured cells (Vero E6—African green monkey kidney cells).

Concerning the field of NMR-based structural biology, the Special Issue contains five original articles in which NMR techniques have been used for structural and dynamical studies of proteins and for elucidating the structural determinants driving molecular recognition mechanisms involved in important biological processes. In the study reported by Lee et al. (contribution 3), NMR spectroscopy is used to describe the coenzyme binding, catalytic turnover, and dynamic behavior of two homologues of Biliverdin reductase B (BLVRB), which is a ubiquitously expressed redox regulator catalyzing the NADPH-dependent reduction of multiple substrates. Ito Y. and co-workers (contribution 5) report the application of NMR methodologies for studying the structure and dynamics of the SH2 domain from the *Drosophila* downstream receptor kinase (Drk). In addition, they investigate, by NMR titration experiments, the interaction of the Drk-SH2 domain with the pY-containing peptide. Timári I. and co-workers (contribution 6) explore, by applying NMR techniques such as ^1^H STD, the interaction of synthesized thiodigalactoside derivatives with human Galectin-3 (hGal-3). This work represents a nice example of an integrated approach in which the NMR techniques were combined with molecular docking methodologies. Muzzioli R. and Gallo A. (contribution 8) define, using an NMR-based multidisciplinary approach, a small library of compounds able to inhibit the protein–protein interaction pathway of ALR (Augmented Liver Regeneration), which is involved in the transport of protein precursors forming disulfides into the mitochondrial intermembrane space. Liu and co-workers (contribution 10) report the three-dimensional structure of Gp49 solved by NMR spectroscopy. The high-resolution NMR structure revealed that the protein consists of six β strands and two helices, with the two helices on the two opposite sides of the β sheet. Moreover, the authors demonstrated that Gp49 is a novel RNA-binding protein that regulates host iron metabolism at the transcriptional level.

I extend my sincere gratitude to all the authors who contributed to this Special Issue with their valuable research. I am also deeply grateful to Dr. Gianluca D’ Abrosca for giving a great contribution during the manuscript revision and to the peer reviewers for their meticulous efforts and constructive feedback, which were essential in guaranteeing the high quality of the published articles. Furthermore, I hope that the articles collected in this Special Issue will serve as a valuable resource and inspire further innovation and application in the field of biomolecular NMR.
